# Leigh-like syndrome with progressive cerebellar atrophy caused by novel *HIBCH* variants

**DOI:** 10.1038/s41439-023-00251-y

**Published:** 2023-08-22

**Authors:** Yoshihiro Taura, Takenori Tozawa, Kenichi Isoda, Satori Hirai, Tomohiro Chiyonobu, Naoko Yano, Takahiro Hayashi, Takeshi Yoshida, Tomoko Iehara

**Affiliations:** 1 Department of Pediatrics, Graduate School of Medical Science, Kyoto Prefectural University of Medicine, Kyoto, Japan; 2 Department of Pediatrics, Ayabe City Hospital, Kyoto, Japan; 3 Department of Pediatrics, Matsushita Memorial Hospital, Osaka, Japan; 4 Department of Pediatric Neurology, Bobath Memorial Hospital, Osaka, Japan; 5 Department of Molecular Diagnostics and Therapeutics, Graduate School of Medical Science, Kyoto Prefectural University of Medicine, Kyoto, Japan; 6 Department of Pediatrics, Kyoto University Graduate School of Medicine, Kyoto, Japan

**Keywords:** Paediatric neurological disorders, Genetic counselling

## Abstract

Pathogenic variants in the *HIBCH* gene cause HIBCH deficiency, leading to mitochondrial disorders associated with valine metabolism. Patients typically present with symptoms such as developmental regression/delay, encephalopathy, hypotonia and dystonia. Brain magnetic resonance imaging (MRI) shows bilateral lesions in the basal ganglia with/without brainstem involvement. Here, we report a case of a Japanese patient with Leigh-like syndrome caused by novel *HIBCH* variants. Long-term follow-up MRI revealed progressive cerebellar atrophy, which expands the phenotypic spectrum of HIBCH deficiency.

The patient was male with nonconsanguineous Japanese parents. He was born at 40 weeks of gestation via normal vaginal delivery following an uneventful pregnancy, and his birth measurements were within the normal ranges. The patient had no family history of neuromuscular disorders or delayed motor development. He gained head control at 4 months of age but was unable to sit up even at 10 months. He experienced poor weight gain from 7 months of age. At 10 months, he developed acute encephalopathy triggered by a viral infection, which led to neurological deterioration. The patient exhibited abnormal eye movements, dystonia of the left upper extremity, and paralysis of the left upper and lower extremities. The ketone body level was 11160 μmol/L. Lactate and pyruvate levels in the blood and cerebrospinal fluid were normal. 3-Hydroxy-isovaleric acid was slightly elevated in urinary organic acid analyses; however, it was classified as a nonspecific finding at that time. Brain magnetic resonance imaging (MRI) revealed bilateral symmetric signal abnormalities in the globus pallidus (Fig. 1A–D), suggesting possible metabolic encephalopathy. The patient was treated with a combination of a vitamin cocktail, carnitine, mannitol, and intravenous immunoglobulin therapy, causing temporary improvement of neurological symptoms. However, the patient subsequently developed various new symptoms, including nystagmus, athetosis, and spastic paraparesis. The abnormal signals in the globus pallidus resolved, but progressive cerebellar atrophy was observed (Fig. 1E–H). The patient acquired gross motor skills only to the point of a walking gait due to hypotonia. At 11 years of age, the patient’s developmental quotient, as assessed using the Kyoto Scale of Psychological Development 2020, was 40. Various tests, including mitochondrial gene point mutation screening, muscle biopsy, mitochondrial respiratory chain enzyme activity in muscle specimens, and ketone enzyme activity levels, yielded normal results.

**Fig. 1:**
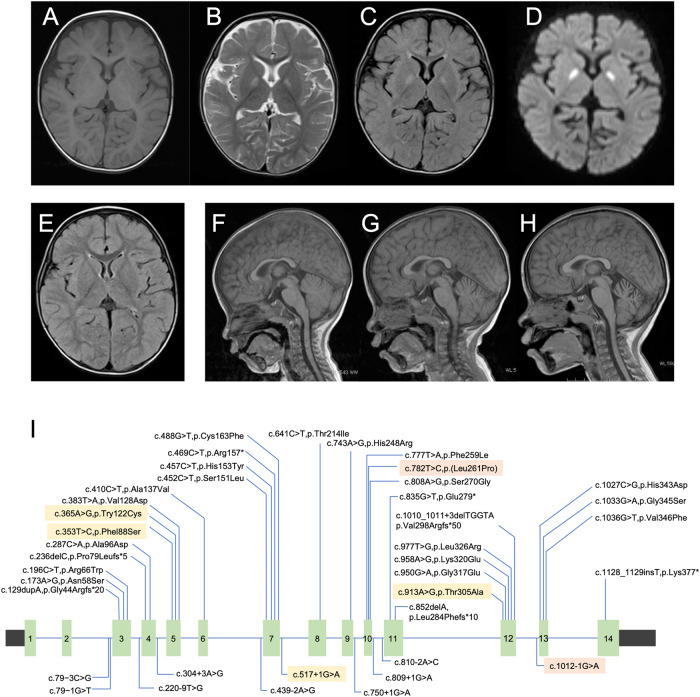
MRI findings and genetic information of the patient. **A**–**D** MRI at ten months showed bilateral symmetric signal abnormalities in the globus pallidus: axial T1-weighted (**A**), T2-weighted (**B**), fluid-attenuated inversion recovery (FLAIR) (**C**), and diffusion-weighted images (**D**). **E** FLAIR image at two years of age showed the disappearance of abnormal signals in the globus pallidus. **F**–**H** Sagittal T1-weighted MRI showing progressive cerebellar atrophy at one year (**F**), two years (**G**), and five years (**H**). **I** Schematic of the *HIBCH* gene with all reported variants labeled. Notes: Red boxes, variants identified in this study; yellow boxes, variants with cerebellar atrophy.

Written informed consent was obtained from the parents in accordance with the Review Board and Ethics Committee of Kyoto University. Whole-exome sequencing (WES) was performed when the patient was 11 years old. Trio-based WES was conducted using the xGen® Exome Research Panel v2 (IDT, Iowa, USA). The captured libraries were sequenced using DNBSEQ-G400 (MGI Tech, Shenzhen, China). WES analysis identified compound heterozygous variants of *HIBCH* that have not yet been reported as pathogenic variants. The first variant was identified in exon 10 [NM_014362.4:c.782 T > C, p.(Leu261Pro)] and was predicted to be deleterious by SIFT (score 0; https://sift.bii.a-star.edu.sg) and disease-causing by MutationTaster (prob 0.999; http://www.mutationtaster.org/). This variant was absent in gnomAD, HGVD, and 8.3KJPN and was moderately conserved across species. The second variant was located in exon 13 (NM_014362.4:c.1012-1 G > A) and was predicted to be deleterious by CADD (score 18.12; https://cadd.gs.washington.edu/) and disease-causing by MutationTaster (prob 0.999; http://www.mutationtaster.org/). This variant was absent in gnomAD, HGVD, and 8.3KJPN. Both variants were confirmed by Sanger sequencing; the c.782 T > C, p.(Leu261Pro) and c.1012-1 G > A variants were maternally and paternally inherited, respectively. Their pathogenicity was evaluated according to the 2015 American College of Medical Genetics and Genomics guidelines^1^. The c.782 T > C, p.(Leu261Pro) and c.1012-1 G > A variants were classified as likely pathogenic and pathogenic, respectively. In this patient, HIBCH deficiency was strongly considered because of the elevation of 3-hydroxy-isovaleric acid in urinary organic acid analyses and Leigh-like syndrome signs on MRI.

3-Hydroxyisobutyryl-CoA hydrolase (HIBCH) deficiency is a rare mitochondrial disorder associated with valine metabolism. Most patients experience delayed motor milestone development in early infancy and develop Leigh/Leigh-like syndrome after a febrile infection or metabolic crisis, leading to secondary regression and a range of neurological symptoms^2^. The movement disorders in this patient are characteristic symptoms of the disease^3,4^. Among them, dystonia, observed from an early onset, is a common symptom, occurring in 71% of patients^3^. Neuroradiological features are similar to those of other causes of Leigh/Leigh-like syndrome, characterized by signal abnormalities in the basal ganglia. In previous cases, the probability of cerebellar atrophy was low at 17%^3^. Table 1 summarizes the characteristics of six reported cases of HIBCH deficiency with cerebellar atrophy, including the present case^3,5^. Five of these six patients had Leigh/Leigh-like syndrome, with dystonia being a typical complication in 5 of 6 patients. Ataxia was present in 3 of 6 patients. All patients except one were unable to walk independently. Patient 2 was considered to have a relatively mild phenotype without Leigh/Leigh-like syndromes, which might be related to residual HIBCH enzyme activity. No deaths occurred during the observation period. Figure 1I shows a schematic of the *HIBCH* gene labeled with all the reported pathogenic variants^6^. There is no hot spot mutation site in the *HIBCH* gene. The variants identified in this study are shown in red boxes. Previously reported variants with cerebellar atrophy are indicated by yellow boxes. The mutation sites of the *HIBCH* gene varied, and there was no correlation between the specific mutation sites and cerebellar atrophy.

**Table 1:** The characteristics of six reported cases of HIBCH deficiency with cerebellum atrophy, including the present case.

Reference	This case	François et al.^3^		Marti-Sanchez L et al.^5^		
Patients No	1	2	3	4	5	6
Pathogenic variants	c.T782C:p.(Leu261Pro)	c.913 A > G p.(Thr305Ala)	c.913 A > G p.(Thr305Ala)	c.365 A > G p.(Tys122Cys)	c.365 A > G p.(Tys122Cys)	c.517+1 G > A
	c.1012-1 G > A	c.913 A > G p.(Thr305Ala)	c.913 A > G p.(Thr305Ala)	c.365 A > G p.(Tys122Cys)	c.365 A > G p.(Tys122Cys)	c.353 T > C p.(Phe188Ser)
Clinical data						
Genetic origin	Japan	No data	No data	Spain	Spain	Spain
Sex	Male	Male	Female	Male	Female	Male
Age of onset	10 months	3 years	2 years	4 years	6 years	4 years
Presentation at onset	Leigh-like syndrome	Exercise-induced paroxysmal dyskinesia	Febrile neurological decompensation meningo-encephalitis	Leigh syndrome	Leigh syndrome	Leigh syndrome
Psychomotor delay	+	+	–	No data	No data	No data
Cognition disorders	+	+	+	No data	No data	No data
Abnormal ocular movements	Nystagmus	–	–	Nystagmus	Nystagmus	–
Movement disorders	Dystonia Ataxia Athetosis	Exercise-induced paroxysmal dyskinesia Dystonia	Dystonia Choreoathetosis Hypokinesia	Ataxia	Dystonia Ataxia Spasticity	Dystonia Spasticity
Other symptoms	Dysarthria Spastic paraparesis Hypotonia Failure to thrive Visual dysfunction	–	Feeding difficulties Visual dysfunction Hypotonia Spasticity Limbs hypertonia Irritability	Ptosis Strabismus	–	Hypotonia Feeding difficulties Visual dysfunction Hearing loss
Reccurrent episodes of neurological involvement	–	No data	No data	–	Acute encephalopathy	Acute encephalopathy and respiratory distress
Seizure	–	–	+	–	–	West syndrome
Evolution	Alive at 11 years	Alive at 11 years	Alive at 9 years	Alive at 18 years	Alive at 15 years	Alive at 8 years
GMFCS score	III	I	IV	IV	IV	No data
Brain imaging data						
Leigh syndrome	+	–	+	+	+	+
Basal ganglia	Globus pallidus	Globus pallidus	Globus pallidus	Globus pallidus	Globus pallidus Candate Putaman	Putaman
White matter involvement	–	–	+	+	–	–
Cerebral atrophy	–	–	–	–	–	–
Cerebellum atrophy	+	+	+	+	+	+
Brain Spectroscopy	No data	No data	↓NAA, lactate peak	No data	No data	No data
Biological data						
Acylcarnitine profile	Normal	No data	Normal	↑C4-OH	↑C4-OH	↑C2 ↑ C3
Plasma amino acids	Normal	Normal	↑Thr, Ala, Gly	Normal	Normal	↑Ala
↑23DH2MB	–	No data	No data	–	–	–
↑3-hydroxy-isovaleric acid	+	No data	No data	No data	No data	No data
Hyperlactatemia	–	–	+	+	–	+
Mitochondrial respiratory chain	Normal	No data	Normal	↓C1	↓C1	↓C1-3
↓PDHc activity	No data	No data	+	No data	No data	+
↓enzymatic activity	No data	No data	+	No data	No data	No data

+ present, – absent, *GMFCS* gross motor function classification system, *NAA* N-Acetylaspartic acid, *23DH2MB* 2,3‐dihydroxy‐2‐methylbutyrate, *PDHc* pyruvate dehydrogenase complex.

To our knowledge, this is the first report of progressive cerebellar atrophy in patients with HIBCH deficiency evaluated using long-term MRI follow-up. Our patient showed no cerebellar atrophy at disease onset; however, progressive atrophy of the cerebellum was revealed over five years. Although there have been only a few reports of HIBCH deficiency with cerebellar atrophy, there may be more cases with follow-up over time that were not detected at the initial diagnosis.

There have been several reports on the usefulness of metabolic analysis^7–9^. Increased C-4 carnitine in the acylcarnitine profile or increased urinary excretion of 2-methyl-2,3-dihydroxybutyrate and 3-hydroxy-isovaleric acid are suggestive findings^3,10^. In our case, C-4 carnitine in the acylcarnitine profile and 2-methyl-2,3-dihydroxybutyrate in urinary organic acid analyses were not elevated, but elevated 3-hydroxy-isovaleric acid levels were detected. In another report, all metabolic analyses yielded negative results despite the severity of the disease^11^. Therefore, in the case of Leigh/Leigh-like syndrome, where movement disorders are the primary symptom and the globus pallidus is involved, a genetic diagnosis using next-generation sequencing should be considered, even if metabolic analyses are negative.

Regarding treatment, some reports have shown improvement in symptoms and imaging findings with a valine-restricted diet^12–14^. In this patient, after the initial rapid worsening of the neurological symptoms, the patient’s manifestations were not exacerbated in the chronic phase, leading the parents to decline dietary treatment after the diagnosis.

In conclusion, HIBCH deficiency is a rare disease with diagnostic challenges due to limited abnormal metabolic studies and various nonspecific movement disorders. However, the presence of bilateral symmetrical signal abnormalities in the globus pallidus during the acute stage and progressive cerebellar atrophy during the chronic stage should raise suspicion of HIBCH deficiency. Considering the potential effect of a valine-restricted diet in the early phase, aggressive genetic analysis should be performed promptly when this syndrome is suspected.

## HGV Database

The relevant data from this Data Report are hosted at the Human Genome Variation Database at 10.6084/m9.figshare.hgv.3315. 10.6084/m9.figshare.hgv.3318.
